# Long-term care insurance pilot exposure and severe-depression risk among older adults with baseline severe IADL impairment: a panel difference-in-difference-in-differences analysis

**DOI:** 10.1186/s12877-026-07861-5

**Published:** 2026-06-23

**Authors:** MingHui Xing, LiXian Wu, ZhaoXue Li, Xiaoyong Yu

**Affiliations:** 1 School of Elderly Care Services and Management, Nanjing University of Chinese Medicine, Nanjing, China; 2 Institute of Elderly Care Services and Management, Nanjing University of Chinese Medicine, Nanjing, China; 3 RuiHua Institute of Elderly Care Services and Management, Nanjing University of Chinese Medicine, Nanjing, China

**Keywords:** Long-term care insurance, Severe depression risk, Difference-in-difference-in-differences, High-care-needs population, CHARLS

## Abstract

**Background:**

In the context of rapid population ageing, long-term care insurance (LTCI) has attracted growing attention for its potential effects on health and well-being. However, most existing studies focus on average changes in depressive symptoms and pay limited attention to severe-depression risk at the upper tail of the mental health distribution. This study examines whether city-level exposure to the LTCI pilot is associated with severe-depression risk among older adults, with particular attention to those with high care needs.

**Methods:**

Using four waves of panel data from the China Health and Retirement Longitudinal Study (CHARLS; 2013, 2015, 2018, and 2020), we constructed a sample of adults aged 60 years and older. The final analytical sample included 27,967 person-year observations from 8,258 respondents, with a mean baseline age of 65.98 years (SD 5.76). We used baseline severe impairment in instrumental activities of daily living (IADL) to identify the high-care-needs population, defined severe depression tail risk using CESD-10 scores > = 16, and estimated intention-to-treat (ITT) associations with city-level LTCI pilot exposure using a difference-in-difference-in-differences (DDD) model.

**Results:**

In the baseline specification, city-level exposure to the LTCI pilot was associated with a higher upper-tail risk of severe depression among older adults with baseline severe IADL impairment. The baseline ITT estimate corresponded to an approximately 12.45-percentage-point higher probability of being in the severe-depression range among this high-care-needs group relative to comparison groups. Additional diagnostics, including exclusion of the 2020 wave, province-level clustering, wild cluster bootstrap inference, and leave-one-treated-city-out checks, supported the positive direction and city-level stability of the estimate. The sex-specific signal appeared stronger among women in exploratory analyses.However, statistical significance weakened under some sample-restriction specifications, and the findings should be interpreted as suggestive rather than conclusive.

**Conclusions:**

The findings suggest that the psychological consequences of LTCI exposure may be more visible in the upper tail of the depression distribution among older adults with high care needs than in average mental-health indicators, with the signal appearing stronger in women in exploratory analyses. Policy evaluation should therefore move beyond mean-based indicators and strengthen needs-matched, psychologically supportive long-term care for vulnerable older adults.

## Introduction

Against the backdrop of rapid population ageing and a steadily rising risk of disability, long-term care has become a central issue in China’s social security system. In 2016, China launched the long-term care insurance (LTCI) pilot programme, marking a substantive new phase in the development of a formal long-term care system. As an important institutional innovation, LTCI is expected not only to ease the caregiving burden faced by older adults with disabilities and their families, but also to improve the allocation of medical resources, care arrangements, and family well-being. Existing studies show that LTCI can reduce hospital utilisation, shorten length of stay, and lower inpatient expenditure and out-of-pocket spending [1–4]. It may also substitute formal care for some acute medical services and informal care, although this substitution appears to vary across income groups [5]. Beyond healthcare utilisation, LTCI has been associated with improvements in self-rated health and cognitive function, reductions in disability, and lower levels of unmet care needs [6–8]. These benefits may extend to the family level by reducing caregiving burden and alleviating adult children’s time, financial, and sleep pressures [8–10]. LTCI should therefore be understood not merely as a financing arrangement for medical care, but as a broader institutional mechanism that shapes caregiving relationships, resource allocation, and the well-being of older adults.

A growing body of research has extended LTCI evaluation from healthcare utilisation to mental health, but much of this work still focuses on average depressive symptoms, subjective well-being, or broad mental-health indicators among older adults [11–17]. Existing heterogeneity analyses commonly compare disabled and non-disabled groups, rural and urban residents, or other broad social categories [13–16]. These studies are important, yet they do not fully address whether LTCI exposure changes the upper tail of the depression distribution among older adults whose care needs are already high at baseline. What remains unclear is not simply whether LTCI exposure changes average depressive symptoms, but whether it shifts the upper-tail risk of severe depression among older adults whose care needs are already high at baseline.

There are theoretical reasons to expect this distributional question to matter. Severe IADL impairment implies greater care dependency, reduced autonomy in daily life, and higher psychological vulnerability. After a pilot programme is introduced, not all high-need older adults necessarily benefit equally from formal coverage: eligibility assessment, service availability, affordability, family coordination, and perceived support may differ across people and cities. For high-care-needs individuals, gaps between institutional coverage and usable, needs-matched support may accumulate as stress rather than immediately improving mental health [18]. For this reason, we hypothesise that policy exposure may be more likely to shift the upper tail of the depression distribution in high-care-needs populations than to alter the mean depressive-symptom level in the overall older population.

Against this background, the literature can be advanced in at least two respects. First, most studies still focus on total CES-D scores or mean levels of depression and therefore pay insufficient attention to tail risks. Second, most heterogeneity analyses use broad categories such as disability status or urban-rural residence, making it difficult to capture gradients in care need intensity. Older adults with high care needs may face the most concentrated psychological risks, so reliance on average effects and coarse grouping may obscure the mental health associations of LTCI pilot exposure for vulnerable populations. We therefore shift the analytical focus from average depressive symptoms to tail risk of severe depression and examine city-level LTCI pilot exposure among older adults with high care needs. Using four waves of CHARLS data (2013, 2015, 2018, and 2020), we define the high-care-needs population using baseline severe IADL impairment, construct a severe depression tail-risk indicator based on CESD-10 thresholds, and estimate a DDD model that compares post-policy changes across treated and control cities as well as across baseline care-need groups.

## Methods

### Data sources and sample construction

This study uses data from the China Health and Retirement Longitudinal Study (CHARLS), a nationally representative longitudinal survey of individuals aged 45 years and older in China. The national baseline survey was conducted in 2011 and covered approximately 10,000 households and 17,500 respondents across 150 counties (districts) and 450 villages or neighbourhood committees [19].

Our main analytical sample includes four waves of CHARLS data: 2013, 2015, 2018, and 2020. Sample selection proceeded in three steps. First, we retained respondents aged 60 years and older. Second, we retained observations with non-missing CESD-10 total scores. Third, to ensure a meaningful pre-post comparison, we retained only individuals observed at least once in the pre-policy period (2013 or 2015) and at least once in the post-policy period (2018 or 2020). The final analytical sample contained 27,967 person-year observations from 8,258 respondents. The 2011 wave was not included in the main specification because baseline severe IADL status was anchored to the earliest pre-policy observation used for the analytic panel structure, and incorporating 2011 would substantially increase sample imbalance and attrition-related complexity across the four-wave main panel. We therefore treated 2011 as an extended-sample robustness exercise rather than as part of the primary specification. For 2020, the CESD-10 total score was reconstructed from the original items using a consistent coding procedure; the reconstructed scores were fully consistent with the analytical data, supporting comparability across waves. The 2020 wave coincided with the COVID-19 period, which may have introduced additional regional shocks to mental health beyond LTCI exposure.

Policy exposure was identified using information on LTCI pilot cities. In 2016, the General Office of the Ministry of Human Resources and Social Security issued the Guiding Opinions on Piloting the Long-Term Care Insurance System, formally launching the pilot programme. Subsequent official responses by the National Healthcare Security Administration indicated that 15 cities and 2 key associated provinces were included in the 2016 pilot [20, 21]. Later official materials listed the original pilot locations as Chengde, Changchun (and subsequently expanded cities in Jilin Province), Qiqihar, Shanghai, Suzhou, Nantong, Ningbo, Anqing, Shangrao, Qingdao (and subsequently expanded cities in Shandong Province), Jingmen, Guangzhou, Chongqing, Chengdu, and Shihezi [22, 23].

Mapping official pilot cities to the CHARLS sample: Given variation in the timing of initiation, implementation pace, and policy maturity across pilot areas, we constructed three policy exposure definitions for identification and robustness purposes. The official first-batch list was broader than the set that can be cleanly observed in CHARLS because not all official pilot locations can be stably matched to city identifiers or supported by sufficient observations in the analytical sample. Treatment A was therefore limited to 12 identifiable pilot cities in our sample: Shanghai, Shangrao, Ningbo, Anqing, Guangzhou, Chengdu, Chengde, Suzhou, Jingmen, Chongqing, Qingdao, and Qiqihar. Treatment B excluded Shanghai and Qingdao, two locations with strong pioneering characteristics, and therefore comprised 10 cities: Shangrao, Ningbo, Anqing, Guangzhou, Chengdu, Chengde, Suzhou, Jingmen, Chongqing, and Qiqihar. Treatment C was the most conservative definition and retained only six cities with relatively stable samples: Shangrao, Ningbo, Guangzhou, Chengdu, Chengde, and Chongqing. Cities outside these definitions were not subjectively excluded to strengthen the findings; rather, they could not be matched with sufficient stability to the CHARLS analytical sample or lacked adequate sample support. Appendix Tables 7 and 8 report cluster counts and treated-city sample counts.

We chose treatment B as the baseline definition because some early pilot cities had especially distinctive implementation paths and policy maturity. Qingdao is described in official materials as one of the earliest Chinese cities to explore LTCI, having established a related system in 2012. Shanghai, although included in the first pilot batch, launched its local pilot measures in January 2017 and displayed particularly strong pioneering and expansionary characteristics. Excluding these two cases therefore yields a more comparable baseline treatment definition. This baseline definition was chosen on substantive institutional grounds rather than because it yielded the strongest statistical significance; indeed, alternative definitions A and C are also reported to show that the sign of the estimate is not driven by a single treatment classification.

### Variables

Outcome variable: The main outcome is upper-tail risk of severe depression. Based on the CESD-10 total score, HighDep16 is coded as 1 if the respondent’s score at time t is 16 or higher, and 0 otherwise. In robustness analyses, we use a more stringent threshold, HighDep20, coded as 1 when the CESD-10 score is 20 or higher. These definitions are intended to capture changes at the upper tail of the mental health risk distribution.$\mathrm{HighDep16}_{\mathrm{it}}\;=\;1\;\mathrm{HighDep20}_{\mathrm{it}}\;=\;1$

In Chinese middle-aged and older populations, the most common screening cut-off for CESD-10 is 10. Latent profile and ROC-based evidence from the 2018 CHARLS sample suggests that 10 is a reasonable threshold for identifying possible depression risk among adults aged 45 years and older, and many CHARLS-based studies use CESD-10 > = 10 to define depressive symptoms. At the same time, previous work has shown substantial variation in optimal thresholds across settings, ranging roughly from 5 to 16 points. Scores of 10–12 are therefore commonly used to screen for possible depression risk, whereas the thresholds of 16 and 20 used here are intended to identify the upper tail of more severe psychological distress rather than to serve as clinical diagnostic cut-offs [20, 21, 24]. We chose CESD-10 > = 16 as the main threshold because the purpose of this study is not to identify general depressive symptoms but to capture the upper-risk tail of severe psychological distress; CESD-10 > = 20 is then used as a stricter robustness threshold.

Key heterogeneity variable: The key heterogeneity dimension is baseline severe impairment in instrumental activities of daily living (IADL). Baseline status is determined using the earliest available pre-policy observation: 2013 is used whenever available, and 2015 is used otherwise. If the number of baseline IADL limitations is two or more, IADL2plusi is coded as 1; otherwise it is coded as 0. This variable identifies the high-care-needs population. Compared with a simple binary indicator of disability, baseline severe IADL impairment better captures variation in the degree of functional limitation and the intensity of potential care needs, thereby providing a more refined basis for examining heterogeneous psychological associations [25–29]. Using a threshold of two or more IADL limitations allows us to distinguish older adults with more substantial functional constraints from those with only mild or isolated difficulty, thereby better approximating a high-care-needs subgroup rather than a broad disability category.

Policy exposure and controls: In the baseline specification, policy exposure is defined by the interaction between pilot-city status and the post-policy period. Let treatedi indicate whether individual i resides in a pilot city and postt indicate the post-policy period (2018 and 2020). Then didit = treatedi x postt. Because CHARLS survey waves are spaced discretely (every two to three years) and the exact month of local implementation varies across pilot cities, we operationalise the post period at the wave level (2018/2020) as an approximation to post-introduction exposure. Given that all 2016 pilot cities launched their programmes between mid-2016 and early 2017 and the next available CHARLS wave is 2018, the 2018 wave captures the post-policy period for all treated cities regardless of within-wave implementation month. The 2020 wave then provides the longest follow-up window currently available. This operationalisation may introduce some measurement error due to within-wave timing variation; because such misclassification in the timing of treatment exposure is likely to be non-differential with respect to the outcome, it would tend to bias the estimates toward the null rather than to inflate them. The baseline analysis uses treatment B, while treatments A and C are used for robustness checks. Control variables include marital status, chronic conditions, medical insurance participation, and pension insurance participation. Importantly, the policy variable captures city-level exposure to LTCI rather than individual enrolment or benefit receipt, so the estimates should be interpreted as average city-level exposure effects rather than individual treatment effects. This design should therefore be interpreted as an intention-to-treat (ITT) framework, capturing the average association with residing in a pilot city after programme introduction, regardless of whether an individual actually enrolled in or used LTCI services.$\mathrm{did}_{\mathrm{it}}\:=\;\mathrm{treated}_{\mathrm{i}}\:\times\:\mathrm{post}_{\mathrm{t}}$

### Empirical model

We estimate a difference-in-difference-in-differences (DDD) model to identify the association between city-level LTCI pilot exposure and severe-depression risk among older adults with high care needs. The coefficient of primary interest is the triple interaction between policy exposure, the post-policy period, and baseline severe IADL impairment. This coefficient captures the incremental post-policy change in severe-depression risk among older adults with baseline severe IADL impairment in pilot cities relative to the corresponding changes in comparison groups.

The baseline DDD specification can be written as:

$$\begin{aligned}\mathrm{HighDep}_{\mathrm{it}}\:&=\:\beta_{1}\mathrm{Did}_{\mathrm{it}}+\:\beta_{2}\:(\mathrm{Post}_{\mathrm{t}}\:\times\:\mathrm{IADL2plus}_{\mathrm{i}})\:\\&+\:\beta_{3}\:(\mathrm{Did}_{\mathrm{it}}\:\times\:\mathrm{IADL2plus}_{\mathrm{i}})\:\\&+\:\mathrm{X}{^{\prime}}_{\mathrm{it}}{\gamma}\:+\:\mu_{\mathrm{i}}\:+\:\tau_{\mathrm{t}}\:+\:\epsilon_{\mathrm{it}},\end{aligned}$$

where β_3_ is the coefficient of primary interest.

The identification strategy relies on the DDD parallel-trends assumption. In the absence of the LTCI pilot, the difference in severe-depression risk between individuals with high care needs and those with lower care needs should have followed similar trends over time in pilot and non-pilot cities. Because baseline severe IADL status is measured before policy implementation, reverse causality from the policy to the third heterogeneity dimension is less likely. Because only two pre-policy waves are available, the event-study results should be viewed as a limited diagnostic rather than strong confirmation of the identifying assumption.

Because the outcome is binary, we estimate linear probability models (LPMs) and cluster standard errors at the city level. The estimation uses 126 city clusters in total; under treatment definitions A, B, and C, the numbers of treated clusters are 12, 10, and 6, respectively, with the remainder serving as control clusters. This specification facilitates direct interpretation of marginal changes in probability in a setting with multiple interaction terms, fixed effects, and alternative policy definitions. All models include individual and year fixed effects.

## Results

### Sample description and baseline characteristics

Table 1 summarizes baseline characteristics of the main analytic sample under treatment definition B. At baseline, the mean age of participants was 65.98 years (SD 5.76), and 51.2% were women. The treated and control groups were broadly comparable at baseline: age, sex composition, marital status, chronic disease prevalence, insurance coverage, baseline CESD-10, and baseline IADL/ADL severity were all similar, with no differences statistically significant at the 5% level. Appendix Table 9 compares baseline characteristics between the IADL2plus = 1 and IADL2plus = 0 groups, showing that the high-care-needs subgroup had higher baseline depressive risk and greater functional limitation.

**Table 1:** Baseline characteristics of the main analytic sample under treatment definition B

variable	overall	treated_B_	control_B_	*p*-value
N	8258	706	7552	
Age	65.98 (5.76)	65.65 (5.38)	66.01 (5.79)	0.091
Female	51.2%	49.2%	51.4%	0.261
Married	83.5%	84.4%	83.4%	0.48
Any chronic disease	73.8%	75.4%	73.6%	0.316
Covered by medical insurance	90.7%	92.2%	90.5%	0.11
Covered by pension	75.0%	74.1%	75.1%	0.562
Baseline CESD-10	8.08 (6.02)	8.06 (5.95)	8.08 (6.03)	0.923
Baseline HighDep16	12.9%	12.9%	13.0%	0.963
Baseline IADL score	0.42 (0.92)	0.39 (0.90)	0.42 (0.93)	0.39
Baseline IADL2plus	11.0%	9.6%	11.1%	0.21
Baseline ADL score	0.38 (0.93)	0.33 (0.87)	0.38 (0.94)	0.187

The table reports baseline characteristics at the earliest pre-policy observation (2013 where available, otherwise 2015). Treatment status follows treatment definition B (pilot cities excluding Shanghai and Qingdao). *P*-values compare treated and control groups using unequal-variance t tests

### Baseline results

Table 2 reports the baseline estimates. In the conventional FE-DID model, the coefficient on didB is 0.0042 for HighDep16 and − 0.0051 for HighDep20, and neither estimate is statistically significant. This suggests that when only the average city-level exposure association is considered, the LTCI pilot is not associated with a significant change in severe-depression risk among older adults overall.

**Table 2:** Association of city-level LTCI pilot exposure with severe depression tail risk in older adults: FE-DID and DDD baseline results

Model	Outcome	Coefficient (SE)	95% CI	*p*-value
FE-DID average	HighDep16	0.0042 (0.0168)	[-0.0288, 0.0372]	0.802
DDD risk gradient	HighDep16	0.1245 (0.0576)	[0.0116, 0.2374]	0.031
FE-DID average	HighDep20	-0.0051 (0.0141)	[-0.0328, 0.0226]	0.717
DDD risk gradient	HighDep20	0.0842 (0.0416)	[0.0026, 0.1657]	0.043

The baseline specification uses treatment B, controls for marital status, chronic diseases, medical insurance participation, and pension insurance participation, and includes individual and year fixed effects. Standard errors are clustered at the city level. The coefficients shown are LPM coefficients, and the coefficient on the core interaction term represents the incremental post-policy association among individuals with baseline severe IADL impairment

When baseline severe IADL impairment is incorporated through the DDD specification, the picture changes. The coefficient on the core triple interaction is 0.1245 (*p* = 0.031) for HighDep16 and 0.0842 (*p* = 0.043) for HighDep20, with both estimates significant at the 5% level in the baseline specification. These results indicate that the association between LTCI pilot exposure and mental health is concentrated not at the sample mean but at the upper tail of the depression distribution among those with greater baseline disability risk. In substantive terms, city-level pilot exposure was associated with a higher probability of being in the severe-depression range among older adults with baseline severe IADL impairment relative to comparison groups. The direction of the findings is consistent across both tail thresholds, although the strength of the evidence is somewhat sensitive to sample specification and estimation choices.

Taken together, the baseline results suggest that city-level LTCI pilot exposure may be more closely reflected in changes in the upper-tail risk of severe depression among older adults with high care needs than in changes in the overall average level of depressive symptoms.

### Consistency across treatment definitions

To assess sensitivity to policy exposure definitions, we re-estimated the main model using treatments A, B, and C, as shown in (Table 3). The direction of the core interaction term is consistent across all three definitions, always indicating a higher severe-depression risk among individuals with baseline severe IADL impairment. Under the broader treatment A definition, the estimate is marginally significant under both tail thresholds. Under the more conservative treatment C definition, significance is stronger for HighDep16 and borderline for HighDep20. These results suggest that the sign of the core association does not depend on a single treatment classification.

**Table 3:** Core results under different treatment definitions

Outcome	Treatment definition	Coefficient (SE)	95% CI	*p*-value
HighDep16	Treatment A	0.1097 (0.0566)	[-0.0012, 0.2206]	0.053
HighDep16	Treatment B	0.1245 (0.0576)	[0.0116, 0.2374]	0.031
HighDep16	Treatment C	0.1342 (0.0499)	[0.0364, 0.2319]	0.007
HighDep20	Treatment A	0.0783 (0.0407)	[-0.0014, 0.1579]	0.054
HighDep20	Treatment B	0.0842 (0.0416)	[0.0026, 0.1657]	0.043
HighDep20	Treatment C	0.0897 (0.0458)	[0.0001, 0.1794]	0.050

The table reports the coefficient on the core interaction term (treat × post × IADL2plus), with clustered standard errors in parentheses and 95% confidence intervals in brackets. Treatment A is the original pilot-city definition; treatment B excludes Shanghai and Qingdao; treatment C is the most conservative pilot-city definition

### Dynamic effects and gender heterogeneity

We further examined the temporal pattern of the city-level policy-exposure association and heterogeneity by sex. Because gender stratification was conducted as a supplementary heterogeneity analysis rather than a pre-registered primary hypothesis test, the sex-specific findings should be interpreted as exploratory. Under the HighDep16 definition, the estimated policy-exposure association is 0.1331 in 2018 (*p* = 0.013) and 0.1237 in 2020 (*p* = 0.080). The point estimate is slightly smaller in 2020 than in 2018, suggesting no clear further intensification over time; the 2020 estimates are less precise and should be interpreted cautiously, particularly given the overlap with the COVID-19 period. In the sex-stratified analysis, the coefficient on the triple interaction is 0.2101 among women (*p* < 0.01) but − 0.0109 among men and statistically insignificant. A pooled formal sex-difference test using the female x treatedB x post x IADL2plus interaction is also positive and statistically significant (0.2101, *p* = 0.043). The observed signal therefore appears to be concentrated primarily among older women with high care needs (Table 4).

**Table 4:** Dynamic effects and gender heterogeneity of the LTCI pilot on severe depression tail risk

Model	Variable	Outcome	Coefficient (SE)	95% CI	*p*-value
Event-study	Pre-trend test: 2013 triple interaction	HighDep16	0.0111 (0.0856)	[-0.1567, 0.1789]	0.897
Event-study	2018 exposure association	HighDep16	0.1331 (0.0538)	[0.0275, 0.2386]	0.013
Event-study	2020 exposure association	HighDep16	0.1237 (0.0706)	[-0.0146, 0.2620]	0.080
Event-study	Pre-trend test: 2013 triple interaction	HighDep20	-0.0453 (0.0799)	[-0.2019, 0.1113]	0.571
Event-study	2018 exposure association	HighDep20	0.1029 (0.0579)	[-0.0106, 0.2164]	0.076
Event-study	2020 exposure association	HighDep20	0.0107 (0.0755)	[-0.1374, 0.1587]	0.887
Female subsample	pilot x post x baseline severe IADL risk	HighDep16	0.2101 (0.0562)	[0.1000, 0.3203]	0.000
Male subsample	pilot x post x baseline severe IADL risk	HighDep16	-0.0109 (0.1142)	[-0.2347, 0.2130]	0.924
Pooled interaction	Formal sex-difference test: female x treatB x post x IADL2plus	HighDep16	0.2101 (0.1040)	[0.0063, 0.4139]	0.043

The pooled formal sex-difference test estimates a four-way interaction between female, treated-city status, the post-policy period, and baseline IADL2plus status in the full sample with individual and year fixed effects and city-clustered standard errors. The test is reported as supplementary exploratory evidence

### Pre-trend and event-study evidence

**Fig. 1:**
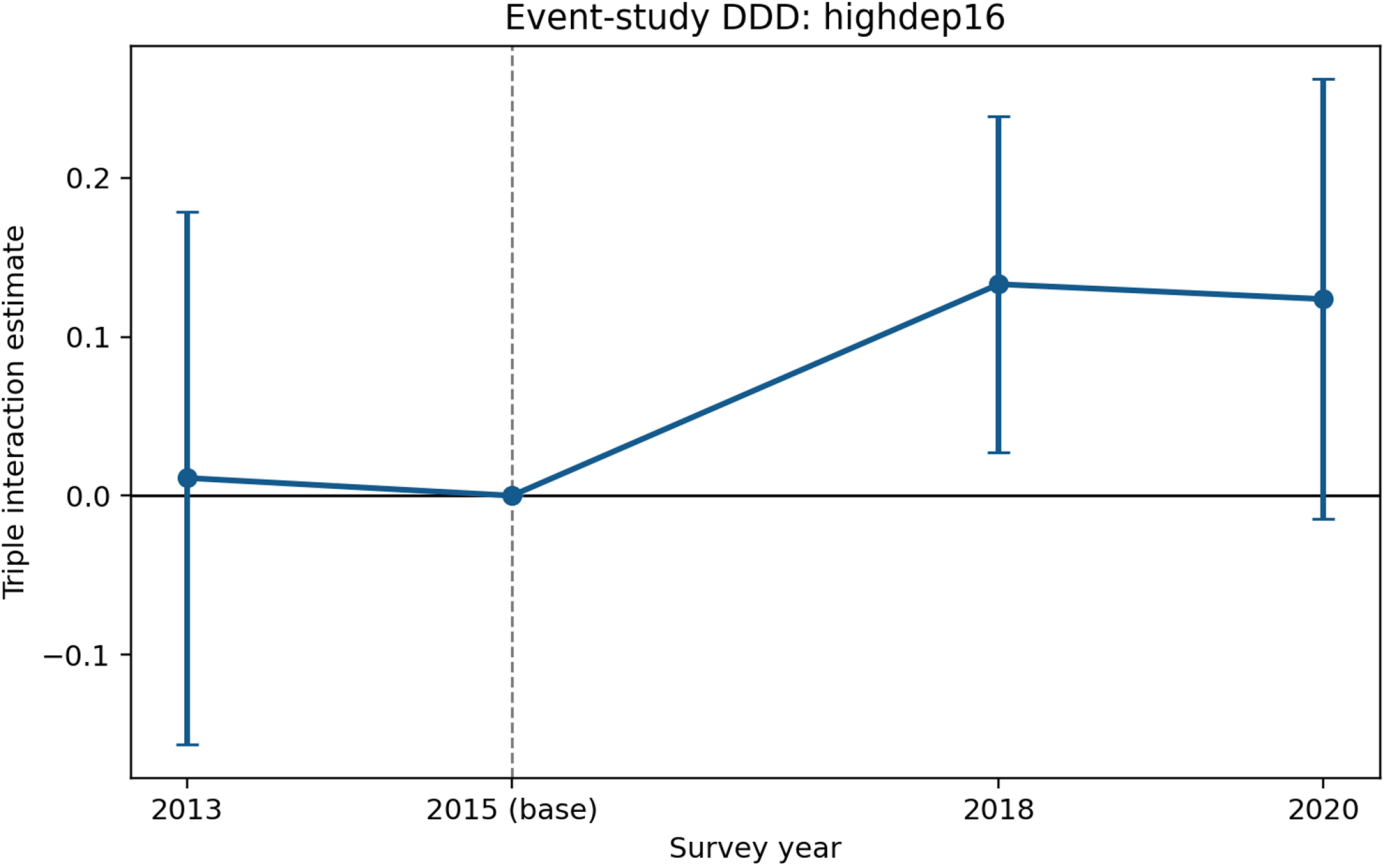
Event-study estimates for the triple interaction under the HighDep16 specification Note: The figure plots the coefficient on the triple interaction term (treated × year × IADL2plus) with 95% confidence intervals, using 2015 as the omitted reference year

**Fig. 2:**
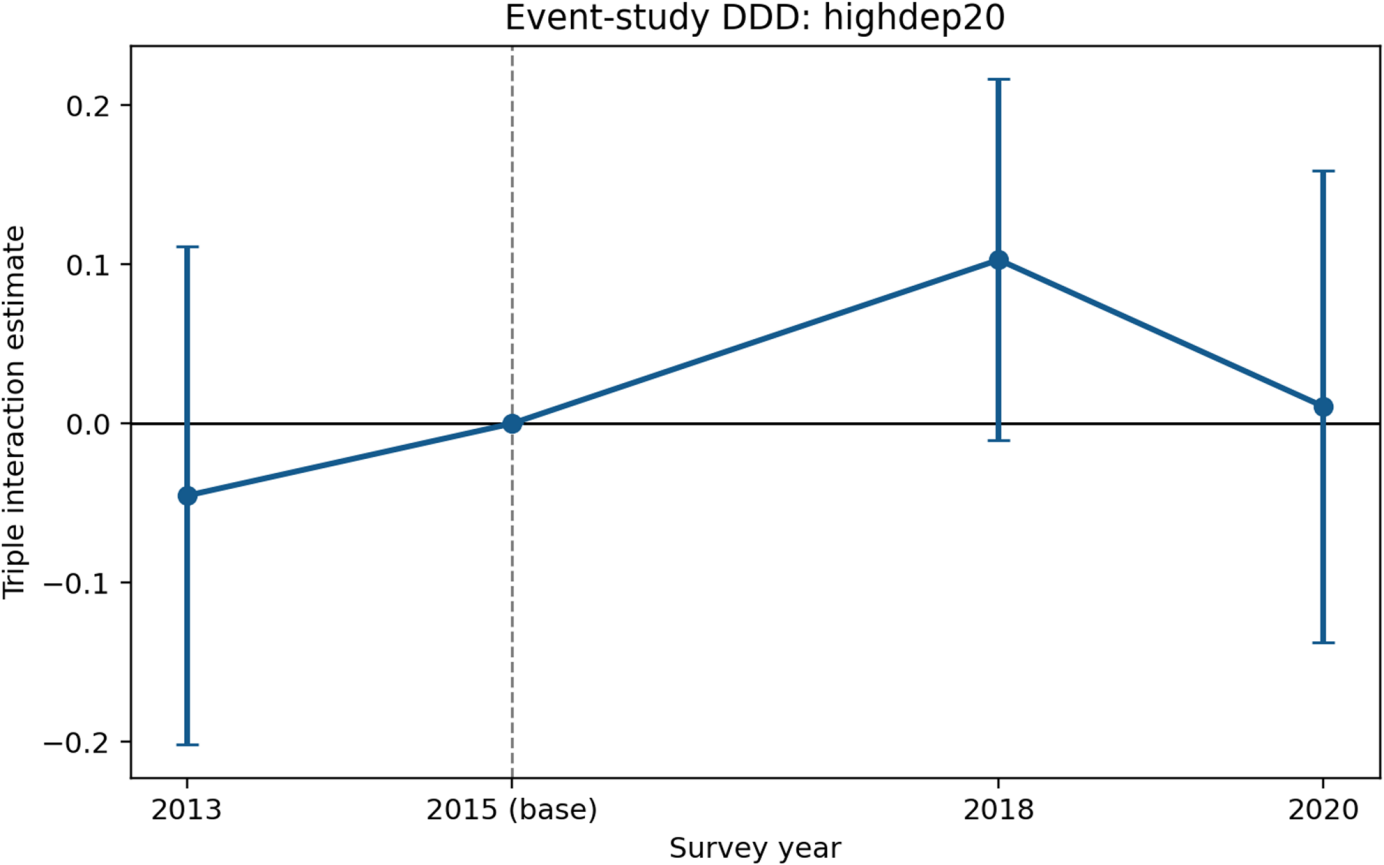
Event-study DDD estimates for severe-depression risk (HighDep20) Note: The figure plots the coefficient on the triple interaction term (treated × year × IADL2plus) with 95% confidence intervals, using 2015 as the omitted reference year

The event-study exercise does not detect evidence of differential pre-trends before policy implementation, although the pre-policy period is short and the test has limited statistical power. The pre-policy triple interaction in 2013 is close to zero for both HighDep16 (0.0111, *p* = 0.897) and HighDep20 (-0.0453, *p* = 0.571). For HighDep16, the triple interaction becomes positive and statistically significant in 2018 (0.1331, *p* = 0.013) and remains positive but less precise in 2020 (0.1237, *p* = 0.080), indicating that the tail-risk association is observed after city-level pilot exposure rather than in the available pre-policy period (Fig. 1). The corresponding event-study results for HighDep20 are presented in Fig. 2; the 2020 estimate for HighDep20 is small and imprecise (0.0107, *p* = 0.887), reinforcing the need for caution in interpreting pandemic-period estimates.

### Robustness checks and interpretation boundaries

We assessed robustness using both the original sample-based checks and additional diagnostics requested during revision. In the original checks, the direction of the core interaction remained stable but statistical significance weakened under several alternative specifications. For HighDep16, the p-value of the core interaction was 0.031 in the main specification and 0.126, 0.367, and 0.123 in the balanced-panel, 2011-extended, and IPW specifications, respectively. For HighDep20, the corresponding p-values were 0.043, 0.413, 0.292, and 0.028, respectively (Table 5). To further evaluate whether the baseline signal was driven by the 2020 COVID-period wave, a particular treated city, or the clustering level, we added four diagnostics: excluding the 2020 wave, clustering at the province level, wild cluster bootstrap inference, and leave-one-treated-city-out re-estimation. These additional checks supported the stability of the positive association: for HighDep16, excluding 2020 remained significant (0.1262, *p* = 0.026), province-clustered inference remained significant (*p* = 0.017), the wild cluster bootstrap p-value was 0.011, and all ten leave-one-treated-city-out estimates remained positive and significant at the 5% level. For HighDep20, all ten leave-one-treated-city-out estimates were positive, with eight significant at the 5% level and nine at the 10% level (Table 6). Taken together, the evidence is not solely dependent on a single treated city or the 2020 wave, although precision remains weaker in the balanced-panel and extended-sample specifications. Full coefficient estimates for the baseline, alternative-treatment, event-study, gender-stratified, and robustness models are reported in Appendix Tables 11-16.

**Table 5:** Core robustness checks

Outcome	Specification	Coefficient (SE)	95% CI	*p*-value
HighDep16	Main specification	0.1245 (0.0576)	[0.0116, 0.2374]	0.031
HighDep16	Balanced panel	0.1175 (0.0767)	[-0.0328, 0.2677]	0.126
HighDep16	2011 extended	0.0663 (0.0734)	[-0.0776, 0.2102]	0.367
HighDep16	IPW	0.1161 (0.0754)	[-0.0316, 0.2639]	0.123
HighDep20	Main specification	0.0842 (0.0416)	[0.0026, 0.1657]	0.043
HighDep20	Balanced panel	0.0561 (0.0686)	[-0.0783, 0.1906]	0.413
HighDep20	2011 extended	0.0604 (0.0572)	[-0.0518, 0.1725]	0.292
HighDep20	IPW	0.0908 (0.0413)	[0.0099, 0.1716]	0.028

The table reports the coefficient on the core interaction term (treat × post × IADL2plus), with clustered standard errors in parentheses and 95% confidence intervals in brackets. The coefficient direction remains positive across all specifications, but statistical significance weakens in the balanced-panel, 2011-extended, and IPW specifications for HighDep16, and in the balanced-panel and 2011-extended specifications for HighDep20. These original robustness checks focus on sample restriction, extended-wave inclusion, and attrition weighting

**Table 6:** Additional robustness diagnostics for the core DDD association

Outcome	Additional diagnostic	Estimate / range	*p*-value / summary
HighDep16	Exclude 2020 wave	0.1262 (0.0565)	0.026
HighDep16	Province-clustered SE	0.1245 (0.0520)	0.017
HighDep16	Wild cluster bootstrap	0.1245	bootstrap *p* = 0.011
HighDep16	Leave-one-treated-city-out	0.1032 to 0.1563	10/10 positive; 10/10 *p* < 0.05
HighDep20	Exclude 2020 wave	0.1252 (0.0390)	0.001
HighDep20	Province-clustered SE	0.0842 (0.0369)	0.023
HighDep20	Wild cluster bootstrap	0.0842	bootstrap *p* = 0.072
HighDep20	Leave-one-treated-city-out	0.0631 to 0.1072	10/10 positive; 8/10 *p* < 0.05; 9/10 *p* < 0.10

The leave-one-treated-city-out diagnostic re-estimates the baseline DDD model after dropping each of the ten Treatment B pilot cities in turn. Wild bootstrap inference uses Rademacher weights at the city-cluster level. These checks are intended to assess sensitivity to pandemic-period observations, clustering level, and single-city influence

## Discussion

Using four waves of CHARLS panel data and a DDD design, this study shows that city-level exposure to the LTCI pilot is associated with a higher probability of being in the severe-depression range among older adults with baseline severe IADL impairment in the baseline specification, whereas no stable change is detected at the average level. In light of the existing literature, this result is better interpreted as evidence of distributional heterogeneity in the psychological associations of LTCI pilot exposure than as a straightforward contradiction of studies reporting average improvements. Additional robustness diagnostics indicate that the positive upper-tail association is not driven by one treated city, by the inclusion of the 2020 wave, or by clustering only at the city level. Research from other domains suggests that policy associations with mental health may first emerge at the tails of the distribution rather than at the mean [30]. Similarly, the mental health effects of pandemic-related experiences are stronger in the upper tail of the depression distribution [31]. Together, these findings suggest that relying exclusively on mean-based indicators may underestimate psychological risks for high-risk groups, while also requiring caution in interpreting city-level ITT estimates.

The result is concentrated among individuals with baseline severe IADL impairment, which is consistent with a well-established gradient relationship between functional impairment and depression. Studies using CHARLS have shown that severe functional impairment is associated with worse long-term depressive trajectories [32], that the risk of depression rises with the degree of functional limitation [25], and that depressive symptoms and IADL/ADL disability may reinforce one another over time [33]. More recent longitudinal evidence also indicates that persistent or worsening functional disability significantly increases later depression risk [34]. In this context, baseline severe IADL impairment serves not only as an indicator of care need intensity, but also as a marker of accumulated psychological vulnerability.

One possible interpretation, which cannot be directly tested with the present data, is that the key issue is not simply whether people with high care needs are nominally covered by the system, but whether institutional arrangements are translated into tangible, needs-matched support. The present study cannot directly observe actual service content, service intensity, quality, affordability, or the degree of fit between received services and individual needs. Previous research shows that unmet long-term care needs are significantly associated with depression and anxiety among both urban and rural older adults, particularly among vulnerable groups [35, 36]. Unmet healthcare needs also increase depressive symptoms, especially among frail older adults [37]. At the same time, other studies suggest that LTCI can improve the overall well-being of older adults and their families [8, 38]. Taken together, our findings are consistent with an interpretation in which formal coverage does not always translate into perceived or usable support for older adults with severe IADL impairment, but this explanation remains speculative and should be tested in future research using enrolment, benefit receipt, service-use, and service-quality data.

These findings carry several policy implications that should be tied closely to the high-care-needs and upper-tail-risk focus of this study. First, LTCI evaluation and implementation should strengthen the precise identification of older adults with substantial baseline IADL impairment rather than relying only on broad age or disability categories [39]. Second, mental-health monitoring should focus on sustained follow-up and severe-depression tail risk, not only general mental-health promotion or average CESD-10 scores [40]. Third, if confirmed in future research, high-care-needs older women may warrant particular attention because the exploratory sex-stratified signal is concentrated in women. Fourth, formal LTCI coverage should be linked to usable, needs-matched, psychologically supportive care, so that assessment, service allocation, family support, community care, and medical services work together rather than remaining parallel systems.

The sex-specific pattern should also be interpreted cautiously. These sex-specific patterns are exploratory and were not the main pre-specified focus of the study, although the pooled formal interaction test indicates that the female-male difference in the core DDD estimate is statistically detectable in this sample. One possible explanation is that older women with high care needs may experience greater caregiving dependence, weaker economic and household bargaining resources, higher emotional burden, and stronger role conflict when formal care arrangements do not fully meet everyday needs. This interpretation is consistent with social-role and vulnerability perspectives, but the present data cannot establish the pathway and future studies should examine between-sex differences with stronger statistical power and richer measures of care arrangements.

The robustness results provide a more nuanced picture than the baseline table alone. On the one hand, the balanced-panel and 2011-extended specifications are less precise, suggesting that the exact magnitude is not fully invariant to sample definition. On the other hand, the additional diagnostics strengthen the credibility of the main signal: excluding the 2020 wave, changing the clustering level, using wild cluster bootstrap inference, and dropping treated cities one at a time all preserve a positive upper-tail association. The current evidence is therefore best read as a stable potential risk signal under city-level pilot exposure, while still stopping short of a definitive individual-level causal claim.

This study also has several strengths. The use of multi-wave CHARLS panel data allows us to track within-individual changes before and after policy implementation more effectively than cross-sectional designs. In addition, extending a standard DID framework to a DDD design using baseline severe IADL impairment provides a more refined way to capture heterogeneity by care need intensity. Finally, shifting attention from average depressive symptoms to severe-depression tail risk makes it possible to detect distributional effects that might otherwise remain hidden.

Several limitations should also be noted. First, although the LTCI pilot creates a quasi-experimental setting, we cannot completely rule out the influence of other contemporaneous regional policies or environmental changes. The 2020 CHARLS wave coincided with the COVID-19 period; pandemic-related stressors may have varied across cities and across high-risk groups, so part of the estimated post-policy association in 2020 may reflect overlapping pandemic-era shocks rather than LTCI exposure alone. Second, the severe-depression risk indicator based on CESD-10 identifies high-risk individuals but cannot fully capture the multidimensional nature of mental health. Third, because the exposure measure reflects city-level policy exposure rather than individual enrolment or benefit receipt, the analysis cannot directly identify effects at the beneficiary level. Fourth, although baseline severe IADL impairment reflects care-need intensity, we do not directly observe the content of care actually received or the degree to which needs were met, so conclusions about mechanisms should remain cautious. Accordingly, the study cannot distinguish between insufficient service intensity, poor service quality, and mismatches between received services and individual care needs. Fifth, variation in actual implementation timing across pilot cities may not be fully captured by the common 2018/2020 post-period coding. Our post-period classification captures all pilot cities at the wave level rather than at the month of local implementation, which introduces some timing imprecision. However, this imprecision is most likely to produce attenuation bias (i.e., toward the null) rather than systematic overestimation, because the misclassification in treatment timing is non-differential with respect to the outcome. Furthermore, the wave-level classification is a practical necessity imposed by the CHARLS data structure, in which mental-health outcomes are observed only at discrete survey waves and exact individual-level exposure start dates are unavailable. Despite this, we cannot rule out the possibility that fine-grained implementation timing heterogeneity could modestly affect the magnitude of the estimated association. Sixth, because policy exposure is defined at the city level and the number of treated clusters is limited, statistical inference may be sensitive to cluster-level sampling variation. Finally, while the direction of the robustness checks is generally stable, statistical significance weakens under some alternative specifications. The findings are therefore best understood as suggestive evidence of a high-risk tail association rather than a definitive or general effect on the mental health of the entire older population. Future work incorporating enrolment status, benefit receipt, service utilisation, service quality, and stronger pre-trend diagnostics would help clarify the pathways through which LTCI exposure is associated with mental health.

## Conclusions

Using CHARLS panel data and a DDD design, this study finds that city-level exposure to the LTCI pilot was not associated with a significant change in severe-depression risk at the average level in the overall sample, but was associated with a higher probability of being in the severe-depression range among older adults with baseline severe IADL impairment. This pattern is consistent with distributional heterogeneity in the mental-health consequences of LTCI exposure. The additional robustness diagnostics support the direction and stability of the upper-tail association, although some sample-restriction checks remain less precise and the results should still be interpreted as city-level ITT associations rather than individual treatment effects. The signal appears stronger in women in exploratory analyses, so the findings should not be generalized uncritically to all high-care-needs older adults. Evaluations of LTCI should therefore move beyond average mental-health indicators and pay greater attention to tail-risk changes among vulnerable groups. From a policy perspective, formal system coverage is not equivalent to effective support; what matters is whether older adults with high care needs can obtain continuous, accessible, needs-matched, and psychologically supportive long-term care services. As LTCI expands and service integration deepens, stronger identification, monitoring, and support for the mental-health risks of vulnerable older adults will be essential.

## Data Availability

The datasets used in the current study are available in the CHARLS repository, https://charls.pku.edu.cn/en/.

## Appendix

Pilot city identification and treatment definitions

**Table 7:** City cluster counts under alternative treatment definitions

treatment_definition	total_city_clusters	treated_city_clusters	control_city_clusters
Treatment A	126	12	114
Treatment B	126	10	116
Treatment C	126	6	120

**Table 8:** Treated-city sample counts in the main analytical sample

City	Respondents	Person-years	IADL2plus respondents	IADL2plus person-years
Shangrao	75	257	11	35
Ningbo	63	219	5	18
Anqin	48	164	5	17
Gaungzhou	78	274	6	20
Chengdu	85	310	7	24
Chengde	64	219	7	23
Suzhou	55	167	1	3
Jingmen	45	155	5	17
Chongqin	148	505	18	60
Qiqihaer	45	158	3	12

**Table 9:** Baseline characteristics by baseline IADL2plus status

Outcome	Coefficient	SE	95% CI	p-value	N	City clusters
HighDep16	0.2101	0.1040	[0.0063, 0.4139]	0.043	27967	126

**Table 10:** Formal sex-difference test

variable	IADL2plus=1	IADL2plus=0	p-value
N	906	7352	nan
Age	67.13 (6.29)	65.84 (5.67)	<0.001
Female	64.9%	49.5%	<0.001
Married	78.9%	84.1%	<0.001
Any chronic disease	85.4%	72.4%	<0.001
Covered by medical insurance	89.8%	90.8%	0.393
Covered by pension	73.1%	75.2%	0.165
Baseline CESD-10	13.18 (6.90)	7.45 (5.59)	<0.001
Baseline HighDep16	36.8%	10.0%	<0.001
Baseline IADL score	2.74 (0.90)	0.13 (0.34)	<0.001
Baseline ADL score	1.84 (1.74)	0.20 (0.55)	<0.001
Definition	Pilot cities		
Treatment A	Shanghai, Shangrao, Ningbo, Anqing, Guangzhou, Chengdu, Chengde, Suzhou, Jingmen, Chongqing, Qingdao, Qiqihar		
Treatment B	Shangrao, Ningbo, Anqing, Guangzhou, Chengdu, Chengde, Suzhou, Jingmen, Chongqing, Qiqihar		
Treatment C	Shangrao, Ningbo, Guangzhou, Chengdu, Chengde, Chongqing		

### Full coefficient output for all reported models

This appendix reports the full coefficient output corresponding to the baseline FE-DID and DDD models, alternative treatment definitions, dynamic/pre-trend models, gender-stratified models, robustness analyses, and event-study specifications.

**Table 11:** Full coefficient output for baseline FE-DID and DDD models

model_name	spec	outcome	coef_name	coef	se	ci_low	ci_high	*p*	nobs	n_id
FE_DID_average	average	highdep16	didB	0.0042	0.0168	-0.0288	0.0372	0.802	27,967	8258
FE_DID_average	average	highdep16	marry	-0.0573	0.0195	-0.0956	-0.0190	0.003	27,967	8258
FE_DID_average	average	highdep16	chronic	0.0138	0.0091	-0.0040	0.0316	0.129	27,967	8258
FE_DID_average	average	highdep16	ins	-0.0132	0.0133	-0.0391	0.0128	0.321	27,967	8258
FE_DID_average	average	highdep16	pension	0.0020	0.0073	-0.0122	0.0163	0.779	27,967	8258
DDD_risk_gradient	risk_gradient	highdep16	triple_iadl2plus	0.1245	0.0576	0.0116	0.2374	0.031	27,967	8258
DDD_risk_gradient	risk_gradient	highdep16	didB	-0.0082	0.0159	-0.0393	0.0229	0.605	27,967	8258
DDD_risk_gradient	risk_gradient	highdep16	post_iadl2plus	-0.0516	0.0184	-0.0875	-0.0156	0.005	27,967	8258
DDD_risk_gradient	risk_gradient	highdep16	marry	-0.0576	0.0195	-0.0958	-0.0193	0.003	27,967	8258
DDD_risk_gradient	risk_gradient	highdep16	chronic	0.0128	0.0090	-0.0049	0.0304	0.156	27,967	8258
DDD_risk_gradient	risk_gradient	highdep16	ins	-0.0133	0.0132	-0.0392	0.0125	0.313	27,967	8258
DDD_risk_gradient	risk_gradient	highdep16	pension	0.0020	0.0073	-0.0123	0.0163	0.784	27,967	8258
FE_DID_average	average	highdep20	didB	-0.0051	0.0141	-0.0328	0.0226	0.717	27,967	8258
FE_DID_average	average	highdep20	marry	-0.0370	0.0123	-0.0612	-0.0128	0.003	27,967	8258
FE_DID_average	average	highdep20	chronic	0.0028	0.0065	-0.0100	0.0156	0.667	27,967	8258
FE_DID_average	average	highdep20	ins	-0.0076	0.0087	-0.0246	0.0094	0.380	27,967	8258
FE_DID_average	average	highdep20	pension	0.0030	0.0049	-0.0066	0.0127	0.537	27,967	8258
DDD_risk_gradient	risk_gradient	highdep20	triple_iadl2plus	0.0842	0.0416	0.0026	0.1657	0.043	27,967	8258
DDD_risk_gradient	risk_gradient	highdep20	didB	-0.0135	0.0135	-0.0399	0.0129	0.316	27,967	8258
DDD_risk_gradient	risk_gradient	highdep20	post_iadl2plus	-0.0335	0.0169	-0.0667	-0.0003	0.048	27,967	8258
DDD_risk_gradient	risk_gradient	highdep20	marry	-0.0371	0.0123	-0.0613	-0.0130	0.003	27,967	8258
DDD_risk_gradient	risk_gradient	highdep20	chronic	0.0021	0.0065	-0.0106	0.0148	0.741	27,967	8258
DDD_risk_gradient	risk_gradient	highdep20	ins	-0.0077	0.0086	-0.0246	0.0091	0.369	27,967	8258
DDD_risk_gradient	risk_gradient	highdep20	pension	0.0030	0.0049	-0.0066	0.0126	0.540	27,967	8258

**Table 12:** Full coefficient output under alternative treatment definitions

model_name	spec	outcome	coef_name	coef	se	ci_low	ci_high	*p*	nobs	n_id
DDD_treatA	treatA	highdep16	triple_iadl2plus	0.1097	0.0566	-0.0012	0.2206	0.053	27,967	8258
DDD_treatA	treatA	highdep16	didA	-0.0053	0.0164	-0.0375	0.0268	0.745	27,967	8258
DDD_treatA	treatA	highdep16	post_iadl2plus	-0.0508	0.0184	-0.0869	-0.0147	0.006	27,967	8258
DDD_treatA	treatA	highdep16	marry	-0.0574	0.0195	-0.0956	-0.0192	0.003	27,967	8258
DDD_treatA	treatA	highdep16	chronic	0.0129	0.0090	-0.0048	0.0305	0.153	27,967	8258
DDD_treatA	treatA	highdep16	ins	-0.0133	0.0132	-0.0391	0.0126	0.314	27,967	8258
DDD_treatA	treatA	highdep16	pension	0.0020	0.0073	-0.0123	0.0163	0.785	27,967	8258
DDD_treatB	treatB	highdep16	triple_iadl2plus	0.1245	0.0576	0.0116	0.2374	0.031	27,967	8258
DDD_treatB	treatB	highdep16	didB	-0.0082	0.0159	-0.0393	0.0229	0.605	27,967	8258
DDD_treatB	treatB	highdep16	post_iadl2plus	-0.0516	0.0184	-0.0875	-0.0156	0.005	27,967	8258
DDD_treatB	treatB	highdep16	marry	-0.0576	0.0195	-0.0958	-0.0193	0.003	27,967	8258
DDD_treatB	treatB	highdep16	chronic	0.0128	0.0090	-0.0049	0.0304	0.156	27,967	8258
DDD_treatB	treatB	highdep16	ins	-0.0133	0.0132	-0.0392	0.0125	0.313	27,967	8258
DDD_treatB	treatB	highdep16	pension	0.0020	0.0073	-0.0123	0.0163	0.784	27,967	8258
DDD_treatC	treatC	highdep16	triple_iadl2plus	0.1342	0.0499	0.0364	0.2319	0.007	27,967	8258
DDD_treatC	treatC	highdep16	didC	-0.0207	0.0190	-0.0579	0.0166	0.277	27,967	8258
DDD_treatC	treatC	highdep16	post_iadl2plus	-0.0501	0.0184	-0.0861	-0.0141	0.006	27,967	8258
DDD_treatC	treatC	highdep16	marry	-0.0575	0.0195	-0.0958	-0.0193	0.003	27,967	8258
DDD_treatC	treatC	highdep16	chronic	0.0127	0.0090	-0.0049	0.0304	0.156	27,967	8258
DDD_treatC	treatC	highdep16	ins	-0.0135	0.0132	-0.0394	0.0124	0.306	27,967	8258
DDD_treatC	treatC	highdep16	pension	0.0020	0.0073	-0.0122	0.0163	0.780	27,967	8258
DDD_treatA	treatA	highdep20	triple_iadl2plus	0.0783	0.0407	-0.0014	0.1579	0.054	27,967	8258
DDD_treatA	treatA	highdep20	didA	-0.0108	0.0129	-0.0360	0.0144	0.401	27,967	8258
DDD_treatA	treatA	highdep20	post_iadl2plus	-0.0334	0.0170	-0.0667	-0.0000	0.050	27,967	8258
DDD_treatA	treatA	highdep20	marry	-0.0371	0.0123	-0.0612	-0.0129	0.003	27,967	8258
DDD_treatA	treatA	highdep20	chronic	0.0022	0.0065	-0.0105	0.0150	0.731	27,967	8258
DDD_treatA	treatA	highdep20	ins	-0.0077	0.0086	-0.0245	0.0092	0.373	27,967	8258
DDD_treatA	treatA	highdep20	pension	0.0030	0.0049	-0.0066	0.0126	0.543	27,967	8258
DDD_treatB	treatB	highdep20	triple_iadl2plus	0.0842	0.0416	0.0026	0.1657	0.043	27,967	8258
DDD_treatB	treatB	highdep20	didB	-0.0135	0.0135	-0.0399	0.0129	0.316	27,967	8258
DDD_treatB	treatB	highdep20	post_iadl2plus	-0.0335	0.0169	-0.0667	-0.0003	0.048	27,967	8258
DDD_treatB	treatB	highdep20	marry	-0.0371	0.0123	-0.0613	-0.0130	0.003	27,967	8258
DDD_treatB	treatB	highdep20	chronic	0.0021	0.0065	-0.0106	0.0148	0.741	27,967	8258
DDD_treatB	treatB	highdep20	ins	-0.0077	0.0086	-0.0246	0.0091	0.369	27,967	8258
DDD_treatB	treatB	highdep20	pension	0.0030	0.0049	-0.0066	0.0126	0.540	27,967	8258
DDD_treatC	treatC	highdep20	triple_iadl2plus	0.0897	0.0458	0.0001	0.1794	0.050	27,967	8258
DDD_treatC	treatC	highdep20	didC	-0.0188	0.0176	-0.0533	0.0156	0.284	27,967	8258
DDD_treatC	treatC	highdep20	post_iadl2plus	-0.0324	0.0167	-0.0652	0.0004	0.053	27,967	8258
DDD_treatC	treatC	highdep20	marry	-0.0372	0.0123	-0.0614	-0.0130	0.003	27,967	8258
DDD_treatC	treatC	highdep20	chronic	0.0022	0.0065	-0.0105	0.0149	0.738	27,967	8258
DDD_treatC	treatC	highdep20	ins	-0.0079	0.0086	-0.0247	0.0090	0.362	27,967	8258
DDD_treatC	treatC	highdep20	pension	0.0030	0.0049	-0.0066	0.0126	0.542	27,967	8258

**Table 13:** Full coefficient output for dynamic/pre-trend models

model_name	spec	outcome	coef_name	coef	se	ci_low	ci_high	*p*	nobs	n_id
DDD_event_study	treatB_event	highdep16	triple_2013	0.0111	0.0856	-0.1567	0.1789	0.897	27,967	8258
DDD_event_study	treatB_event	highdep16	triple_2018	0.1331	0.0538	0.0275	0.2386	0.013	27,967	8258
DDD_event_study	treatB_event	highdep16	triple_2020	0.1237	0.0706	-0.0146	0.2620	0.080	27,967	8258
DDD_event_study	treatB_event	highdep16	treat_2013	-0.0065	0.0233	-0.0522	0.0393	0.781	27,967	8258
DDD_event_study	treatB_event	highdep16	treat_2018	-0.0139	0.0156	-0.0446	0.0167	0.373	27,967	8258
DDD_event_study	treatB_event	highdep16	treat_2020	-0.0070	0.0281	-0.0620	0.0480	0.803	27,967	8258
DDD_event_study	treatB_event	highdep16	iadl_2013	-0.0066	0.0308	-0.0669	0.0537	0.830	27,967	8258
DDD_event_study	treatB_event	highdep16	iadl_2018	-0.0546	0.0223	-0.0984	-0.0108	0.015	27,967	8258
DDD_event_study	treatB_event	highdep16	iadl_2020	-0.0539	0.0305	-0.1137	0.0059	0.077	27,967	8258
DDD_event_study	treatB_event	highdep16	marry	-0.0575	0.0195	-0.0957	-0.0193	0.003	27,967	8258
DDD_event_study	treatB_event	highdep16	chronic	0.0128	0.0090	-0.0048	0.0305	0.154	27,967	8258
DDD_event_study	treatB_event	highdep16	ins	-0.0134	0.0132	-0.0392	0.0125	0.312	27,967	8258
DDD_event_study	treatB_event	highdep16	pension	0.0021	0.0072	-0.0121	0.0163	0.775	27,967	8258
DDD_event_study	treatB_event	highdep20	triple_2013	-0.0453	0.0799	-0.2019	0.1113	0.571	27,967	8258
DDD_event_study	treatB_event	highdep20	triple_2018	0.1029	0.0579	-0.0106	0.2164	0.076	27,967	8258
DDD_event_study	treatB_event	highdep20	triple_2020	0.0107	0.0755	-0.1374	0.1587	0.887	27,967	8258
DDD_event_study	treatB_event	highdep20	treat_2013	-0.0139	0.0154	-0.0440	0.0162	0.364	27,967	8258
DDD_event_study	treatB_event	highdep20	treat_2018	-0.0285	0.0154	-0.0587	0.0018	0.065	27,967	8258
DDD_event_study	treatB_event	highdep20	treat_2020	-0.0074	0.0215	-0.0496	0.0347	0.729	27,967	8258
DDD_event_study	treatB_event	highdep20	iadl_2013	-0.0220	0.0305	-0.0818	0.0378	0.471	27,967	8258
DDD_event_study	treatB_event	highdep20	iadl_2018	-0.0451	0.0220	-0.0882	-0.0021	0.040	27,967	8258
DDD_event_study	treatB_event	highdep20	iadl_2020	-0.0390	0.0305	-0.0987	0.0208	0.201	27,967	8258
DDD_event_study	treatB_event	highdep20	marry	-0.0372	0.0123	-0.0614	-0.0131	0.003	27,967	8258
DDD_event_study	treatB_event	highdep20	chronic	0.0023	0.0065	-0.0103	0.0150	0.718	27,967	8258
DDD_event_study	treatB_event	highdep20	ins	-0.0078	0.0086	-0.0247	0.0090	0.363	27,967	8258
DDD_event_study	treatB_event	highdep20	pension	0.0032	0.0049	-0.0064	0.0128	0.514	27,967	8258

**Table 14:** Full coefficient output for gender-stratified models

model_name	spec	outcome	coef_name	coef	se	ci_low	ci_high	*p*	nobs	n_id
DDD_female	female	highdep16	triple_iadl2plus	0.2101	0.0562	0.1000	0.3203	0.000	14,160	4226
DDD_female	female	highdep16	didB	0.0024	0.0249	-0.0464	0.0511	0.924	14,160	4226
DDD_female	female	highdep16	post_iadl2plus	-0.0676	0.0240	-0.1147	-0.0205	0.005	14,160	4226
DDD_female	female	highdep16	marry	-0.0626	0.0233	-0.1082	-0.0169	0.007	14,160	4226
DDD_female	female	highdep16	chronic	0.0281	0.0149	-0.0011	0.0574	0.060	14,160	4226
DDD_female	female	highdep16	ins	-0.0114	0.0175	-0.0457	0.0228	0.514	14,160	4226
DDD_female	female	highdep16	pension	0.0060	0.0103	-0.0142	0.0262	0.559	14,160	4226
DDD_male	male	highdep16	triple_iadl2plus	-0.0109	0.1142	-0.2347	0.2130	0.924	13,807	4032
DDD_male	male	highdep16	didB	-0.0165	0.0186	-0.0529	0.0199	0.375	13,807	4032
DDD_male	male	highdep16	post_iadl2plus	-0.0280	0.0335	-0.0938	0.0377	0.403	13,807	4032
DDD_male	male	highdep16	marry	-0.0416	0.0292	-0.0988	0.0156	0.154	13,807	4032
DDD_male	male	highdep16	chronic	-0.0010	0.0095	-0.0195	0.0176	0.919	13,807	4032
DDD_male	male	highdep16	ins	-0.0163	0.0170	-0.0497	0.0170	0.337	13,807	4032
DDD_male	male	highdep16	pension	-0.0025	0.0096	-0.0213	0.0163	0.794	13,807	4032

**Table 15:** Full coefficient output for robustness models

model_name	spec	outcome	coef_name	coef	se	ci_low	ci_high	*p*	nobs	n_id
DDD_main	main	highdep16	triple_iadl2plus	0.1245	0.0576	0.0116	0.2374	0.031	27,967	8258
DDD_main	main	highdep16	didB	-0.0082	0.0159	-0.0393	0.0229	0.605	27,967	8258
DDD_main	main	highdep16	post_iadl2plus	-0.0516	0.0184	-0.0875	-0.0156	0.005	27,967	8258
DDD_main	main	highdep16	marry	-0.0576	0.0195	-0.0958	-0.0193	0.003	27,967	8258
DDD_main	main	highdep16	chronic	0.0128	0.0090	-0.0049	0.0304	0.156	27,967	8258
DDD_main	main	highdep16	ins	-0.0133	0.0132	-0.0392	0.0125	0.313	27,967	8258
DDD_main	main	highdep16	pension	0.0020	0.0073	-0.0123	0.0163	0.784	27,967	8258
DDD_balanced4waves	balanced4waves	highdep16	triple_iadl2plus	0.1175	0.0767	-0.0328	0.2677	0.126	16,064	4016
DDD_balanced4waves	balanced4waves	highdep16	didB	-0.0062	0.0209	-0.0472	0.0348	0.766	16,064	4016
DDD_balanced4waves	balanced4waves	highdep16	post_iadl2plus	-0.0206	0.0244	-0.0685	0.0273	0.400	16,064	4016
DDD_balanced4waves	balanced4waves	highdep16	marry	-0.0475	0.0229	-0.0925	-0.0026	0.038	16,064	4016
DDD_balanced4waves	balanced4waves	highdep16	chronic	0.0203	0.0104	-0.0001	0.0407	0.051	16,064	4016
DDD_balanced4waves	balanced4waves	highdep16	ins	-0.0058	0.0195	-0.0441	0.0325	0.766	16,064	4016
DDD_balanced4waves	balanced4waves	highdep16	pension	0.0086	0.0099	-0.0107	0.0279	0.383	16,064	4016
DDD_expanded2011	expanded2011	highdep16	triple_iadl2plus	0.0663	0.0734	-0.0776	0.2102	0.367	32,686	8359
DDD_expanded2011	expanded2011	highdep16	didB	-0.0036	0.0187	-0.0402	0.0330	0.849	32,686	8359
DDD_expanded2011	expanded2011	highdep16	post_iadl2plus	-0.0696	0.0184	-0.1057	-0.0336	0.000	32,686	8359
DDD_expanded2011	expanded2011	highdep16	marry	-0.0580	0.0178	-0.0930	-0.0230	0.001	32,686	8359
DDD_expanded2011	expanded2011	highdep16	chronic	0.0138	0.0086	-0.0030	0.0306	0.108	32,686	8359
DDD_expanded2011	expanded2011	highdep16	ins	-0.0119	0.0115	-0.0345	0.0107	0.302	32,686	8359
DDD_expanded2011	expanded2011	highdep16	pension	-0.0024	0.0059	-0.0139	0.0091	0.686	32,686	8359
DDD_ipw_main	ipw_main	highdep16	triple_iadl2plus	0.1161	0.0754	-0.0316	0.2639	0.123	27,967	8258
DDD_ipw_main	ipw_main	highdep16	didB	-0.0140	0.0162	-0.0457	0.0177	0.388	27,967	8258
DDD_ipw_main	ipw_main	highdep16	post_iadl2plus	-0.0612	0.0192	-0.0988	-0.0236	0.001	27,967	8258
DDD_ipw_main	ipw_main	highdep16	marry	-0.0526	0.0201	-0.0921	-0.0131	0.009	27,967	8258
DDD_ipw_main	ipw_main	highdep16	chronic	0.0111	0.0096	-0.0077	0.0298	0.247	27,967	8258
DDD_ipw_main	ipw_main	highdep16	ins	-0.0146	0.0128	-0.0397	0.0106	0.257	27,967	8258
DDD_ipw_main	ipw_main	highdep16	pension	0.0030	0.0078	-0.0123	0.0183	0.698	27,967	8258
DDD_main	main	highdep20	triple_iadl2plus	0.0842	0.0416	0.0026	0.1657	0.043	27,967	8258
DDD_main	main	highdep20	didB	-0.0135	0.0135	-0.0399	0.0129	0.316	27,967	8258
DDD_main	main	highdep20	post_iadl2plus	-0.0335	0.0169	-0.0667	-0.0003	0.048	27,967	8258
DDD_main	main	highdep20	marry	-0.0371	0.0123	-0.0613	-0.0130	0.003	27,967	8258
DDD_main	main	highdep20	chronic	0.0021	0.0065	-0.0106	0.0148	0.741	27,967	8258
DDD_main	main	highdep20	ins	-0.0077	0.0086	-0.0246	0.0091	0.369	27,967	8258
DDD_main	main	highdep20	pension	0.0030	0.0049	-0.0066	0.0126	0.540	27,967	8258
DDD_balanced4waves	balanced4waves	highdep20	triple_iadl2plus	0.0561	0.0686	-0.0783	0.1906	0.413	16,064	4016
DDD_balanced4waves	balanced4waves	highdep20	didB	-0.0152	0.0145	-0.0437	0.0132	0.293	16,064	4016
DDD_balanced4waves	balanced4waves	highdep20	post_iadl2plus	-0.0019	0.0231	-0.0472	0.0434	0.935	16,064	4016
DDD_balanced4waves	balanced4waves	highdep20	marry	-0.0319	0.0152	-0.0617	-0.0022	0.035	16,064	4016
DDD_balanced4waves	balanced4waves	highdep20	chronic	-0.0006	0.0072	-0.0147	0.0135	0.931	16,064	4016
DDD_balanced4waves	balanced4waves	highdep20	ins	-0.0036	0.0142	-0.0314	0.0243	0.801	16,064	4016
DDD_balanced4waves	balanced4waves	highdep20	pension	0.0060	0.0059	-0.0055	0.0175	0.305	16,064	4016
DDD_expanded2011	expanded2011	highdep20	triple_iadl2plus	0.0604	0.0572	-0.0518	0.1725	0.292	32,686	8359
DDD_expanded2011	expanded2011	highdep20	didB	-0.0137	0.0130	-0.0392	0.0118	0.293	32,686	8359
DDD_expanded2011	expanded2011	highdep20	post_iadl2plus	-0.0434	0.0138	-0.0705	-0.0164	0.002	32,686	8359
DDD_expanded2011	expanded2011	highdep20	marry	-0.0297	0.0126	-0.0544	-0.0050	0.018	32,686	8359
DDD_expanded2011	expanded2011	highdep20	chronic	0.0028	0.0059	-0.0089	0.0144	0.638	32,686	8359
DDD_expanded2011	expanded2011	highdep20	ins	-0.0096	0.0079	-0.0251	0.0059	0.226	32,686	8359
DDD_expanded2011	expanded2011	highdep20	pension	0.0000	0.0041	-0.0081	0.0082	0.995	32,686	8359
DDD_ipw_main	ipw_main	highdep20	triple_iadl2plus	0.0908	0.0413	0.0099	0.1716	0.028	27,967	8258
DDD_ipw_main	ipw_main	highdep20	didB	-0.0152	0.0150	-0.0446	0.0141	0.309	27,967	8258
DDD_ipw_main	ipw_main	highdep20	post_iadl2plus	-0.0358	0.0181	-0.0714	-0.0003	0.048	27,967	8258
DDD_ipw_main	ipw_main	highdep20	marry	-0.0370	0.0130	-0.0626	-0.0114	0.005	27,967	8258
DDD_ipw_main	ipw_main	highdep20	chronic	-0.0002	0.0069	-0.0137	0.0134	0.981	27,967	8258
DDD_ipw_main	ipw_main	highdep20	ins	-0.0090	0.0090	-0.0267	0.0086	0.316	27,967	8258
DDD_ipw_main	ipw_main	highdep20	pension	0.0045	0.0052	-0.0057	0.0148	0.388	27,967	8258

**Table 16:** Full coefficient output for event-study specifications

model_name	spec	outcome	coef_name	coef	se	ci_low	ci_high	*p*	nobs	n_id
DDD_event_highdep16	treatB_event	highdep16	triple_2013	0.0111	0.0856	-0.1567	0.1789	0.897	27,967	8258
DDD_event_highdep16	treatB_event	highdep16	triple_2018	0.1331	0.0538	0.0275	0.2386	0.013	27,967	8258
DDD_event_highdep16	treatB_event	highdep16	triple_2020	0.1237	0.0706	-0.0146	0.2620	0.080	27,967	8258
DDD_event_highdep16	treatB_event	highdep16	treat_2013	-0.0065	0.0233	-0.0522	0.0393	0.781	27,967	8258
DDD_event_highdep16	treatB_event	highdep16	treat_2018	-0.0139	0.0156	-0.0446	0.0167	0.373	27,967	8258
DDD_event_highdep16	treatB_event	highdep16	treat_2020	-0.0070	0.0281	-0.0620	0.0480	0.803	27,967	8258
DDD_event_highdep16	treatB_event	highdep16	iadl_2013	-0.0066	0.0308	-0.0669	0.0537	0.830	27,967	8258
DDD_event_highdep16	treatB_event	highdep16	iadl_2018	-0.0546	0.0223	-0.0984	-0.0108	0.015	27,967	8258
DDD_event_highdep16	treatB_event	highdep16	iadl_2020	-0.0539	0.0305	-0.1137	0.0059	0.077	27,967	8258
DDD_event_highdep16	treatB_event	highdep16	marry	-0.0575	0.0195	-0.0957	-0.0193	0.003	27,967	8258
DDD_event_highdep16	treatB_event	highdep16	chronic	0.0128	0.0090	-0.0048	0.0305	0.154	27,967	8258
DDD_event_highdep16	treatB_event	highdep16	ins	-0.0134	0.0132	-0.0392	0.0125	0.312	27,967	8258
DDD_event_highdep16	treatB_event	highdep16	pension	0.0021	0.0072	-0.0121	0.0163	0.775	27,967	8258
DDD_event_highdep20	treatB_event	highdep20	triple_2013	-0.0453	0.0799	-0.2019	0.1113	0.571	27,967	8258
DDD_event_highdep20	treatB_event	highdep20	triple_2018	0.1029	0.0579	-0.0106	0.2164	0.076	27,967	8258
DDD_event_highdep20	treatB_event	highdep20	triple_2020	0.0107	0.0755	-0.1374	0.1587	0.887	27,967	8258
DDD_event_highdep20	treatB_event	highdep20	treat_2013	-0.0139	0.0154	-0.0440	0.0162	0.364	27,967	8258
DDD_event_highdep20	treatB_event	highdep20	treat_2018	-0.0285	0.0154	-0.0587	0.0018	0.065	27,967	8258
DDD_event_highdep20	treatB_event	highdep20	treat_2020	-0.0074	0.0215	-0.0496	0.0347	0.729	27,967	8258
DDD_event_highdep20	treatB_event	highdep20	iadl_2013	-0.0220	0.0305	-0.0818	0.0378	0.471	27,967	8258
DDD_event_highdep20	treatB_event	highdep20	iadl_2018	-0.0451	0.0220	-0.0882	-0.0021	0.040	27,967	8258
DDD_event_highdep20	treatB_event	highdep20	iadl_2020	-0.0390	0.0305	-0.0987	0.0208	0.201	27,967	8258
DDD_event_highdep20	treatB_event	highdep20	marry	-0.0372	0.0123	-0.0614	-0.0131	0.003	27,967	8258
DDD_event_highdep20	treatB_event	highdep20	chronic	0.0023	0.0065	-0.0103	0.0150	0.718	27,967	8258
DDD_event_highdep20	treatB_event	highdep20	ins	-0.0078	0.0086	-0.0247	0.0090	0.363	27,967	8258
DDD_event_highdep20	treatB_event	highdep20	pension	0.0032	0.0049	-0.0064	0.0128	0.514	27,967	8258

## Abbreviations

LTCI: Long-term care insurance
CHARLS: China Health and Retirement Longitudinal Study
IADL: Instrumental activities of daily living
ADL: Activities of daily living
CESD-10: 10-item Center for Epidemiologic Studies Depression Scale
DDD: Difference-in-difference-in-differences
DID: Difference-in-differences
LPM: Linear probability model
IPW: Inverse probability weighting
CI: Confidence interval
